# Transactions of the Medical Society of the State of New York

**Published:** 1843-04-29

**Authors:** 


*Transactions of the Medical Society of the State of New York.* Vol. V. Part III. 1843. Albany, 1843. 8vo.

The present part of the fifth volume of the Transactions contains the Annual Address, by Dr. Taylor, the President of the Society; Homœopathy Illustrated, by Dr. T. W. Blatchford; and Insanity, its causes, pathology and treatment, by Dr. C. B. Coventry, of Geneva College. The subject which Dr. Taylor has chosen is that of typhoid fever; it is treated as a sensible practical man would treat it, avoiding all the intricacies of the subject. At this moment, when quackery is in such general repute, the address of Dr. Blatchford is opportune. To contend successfully against popular delusions the profession should understand, or endeavour to understand them perfectly—for sometimes they are, we confess, above all comprehension. Dr. B. has sketched the principal doctrines of homœopathy fairly and successfully, and exposed ably its fallacies. We would recommend the perusal of Dr. Blatchford's address to the profession generally. The subject of Dr. Coventry's contribution has of late received so much attention from us, that we cannot do more at present than say it is a creditable performance.

The appendix to this *brochure* contains the Report of the Committee on Medical Societies, &c., of the New York House of Assembly in 1842, on the application of the Thomsonians to admit them to a legal equality with other physicians in that State. The report is adverse to granting the petition, inasmuch as the applicants do not comply with the provisions of the statute regulating medical instruction. In the Senate the same committee reported unanimously against "levelling all distinctions between learned and unlearned physicians," and as a substitute to the bill under consideration, proposed one in which the qualifications for a license were raised as follows:

§ 1. No person shall be admitted to an examination as a physician or surgeon, unless he shall have studied seven years in the office of a practising physician or surgeon, deducting therefrom the time he shall have pursued classical studies and attended medical lectures, not exceeding in all four years.

§ 2. No person shall receive the degree of doctor of medicine until he shall have been for three years a licensed physician; or until he shall have been licensed as a physician and surgeon, and subsequent thereto shall have spent six months as a pupil in some of the public hospitals of this State.

Unfortunately, however, on February 11th, 1843, the Select Committee of the Assembly reported a bill repealing the laws restricting the practice, accompanied with a report of the most absurd and radical character.

